# 90 Higher Initial Formula for Resuscitation After Severe Burn Injury Means Higher 24-Hour Volumes

**DOI:** 10.1093/jbcr/irad045.063

**Published:** 2023-05-15

**Authors:** Julie Rizzo, Elsa Coates, Maria Serio-Melvin, James Aden, Jonathan Stallings, Kevin Foster, Kareem AbdelFattah, Tam Pham, Jose Salinas

**Affiliations:** Brooke Army Medical Center, La Vernia, Texas; United States Army Institute of Surgical Research, San Antonio, Texas; U.S. Army Institute of Surgical Research, Ft. Sam Houston, Texas; Brooke Army Medical Center, San Antonio, Texas; United States Army Institute of Surgical Research, San Antonio, Texas; AZ Burn Center at Valleywise Health, Cave Creek, Arizona; UT Southwestern, Dallas, Texas; University of Washington, Seattle, Washington; United States Institute of Surgical Research, San Antonio, Texas; Brooke Army Medical Center, La Vernia, Texas; United States Army Institute of Surgical Research, San Antonio, Texas; U.S. Army Institute of Surgical Research, Ft. Sam Houston, Texas; Brooke Army Medical Center, San Antonio, Texas; United States Army Institute of Surgical Research, San Antonio, Texas; AZ Burn Center at Valleywise Health, Cave Creek, Arizona; UT Southwestern, Dallas, Texas; University of Washington, Seattle, Washington; United States Institute of Surgical Research, San Antonio, Texas; Brooke Army Medical Center, La Vernia, Texas; United States Army Institute of Surgical Research, San Antonio, Texas; U.S. Army Institute of Surgical Research, Ft. Sam Houston, Texas; Brooke Army Medical Center, San Antonio, Texas; United States Army Institute of Surgical Research, San Antonio, Texas; AZ Burn Center at Valleywise Health, Cave Creek, Arizona; UT Southwestern, Dallas, Texas; University of Washington, Seattle, Washington; United States Institute of Surgical Research, San Antonio, Texas; Brooke Army Medical Center, La Vernia, Texas; United States Army Institute of Surgical Research, San Antonio, Texas; U.S. Army Institute of Surgical Research, Ft. Sam Houston, Texas; Brooke Army Medical Center, San Antonio, Texas; United States Army Institute of Surgical Research, San Antonio, Texas; AZ Burn Center at Valleywise Health, Cave Creek, Arizona; UT Southwestern, Dallas, Texas; University of Washington, Seattle, Washington; United States Institute of Surgical Research, San Antonio, Texas; Brooke Army Medical Center, La Vernia, Texas; United States Army Institute of Surgical Research, San Antonio, Texas; U.S. Army Institute of Surgical Research, Ft. Sam Houston, Texas; Brooke Army Medical Center, San Antonio, Texas; United States Army Institute of Surgical Research, San Antonio, Texas; AZ Burn Center at Valleywise Health, Cave Creek, Arizona; UT Southwestern, Dallas, Texas; University of Washington, Seattle, Washington; United States Institute of Surgical Research, San Antonio, Texas; Brooke Army Medical Center, La Vernia, Texas; United States Army Institute of Surgical Research, San Antonio, Texas; U.S. Army Institute of Surgical Research, Ft. Sam Houston, Texas; Brooke Army Medical Center, San Antonio, Texas; United States Army Institute of Surgical Research, San Antonio, Texas; AZ Burn Center at Valleywise Health, Cave Creek, Arizona; UT Southwestern, Dallas, Texas; University of Washington, Seattle, Washington; United States Institute of Surgical Research, San Antonio, Texas; Brooke Army Medical Center, La Vernia, Texas; United States Army Institute of Surgical Research, San Antonio, Texas; U.S. Army Institute of Surgical Research, Ft. Sam Houston, Texas; Brooke Army Medical Center, San Antonio, Texas; United States Army Institute of Surgical Research, San Antonio, Texas; AZ Burn Center at Valleywise Health, Cave Creek, Arizona; UT Southwestern, Dallas, Texas; University of Washington, Seattle, Washington; United States Institute of Surgical Research, San Antonio, Texas; Brooke Army Medical Center, La Vernia, Texas; United States Army Institute of Surgical Research, San Antonio, Texas; U.S. Army Institute of Surgical Research, Ft. Sam Houston, Texas; Brooke Army Medical Center, San Antonio, Texas; United States Army Institute of Surgical Research, San Antonio, Texas; AZ Burn Center at Valleywise Health, Cave Creek, Arizona; UT Southwestern, Dallas, Texas; University of Washington, Seattle, Washington; United States Institute of Surgical Research, San Antonio, Texas; Brooke Army Medical Center, La Vernia, Texas; United States Army Institute of Surgical Research, San Antonio, Texas; U.S. Army Institute of Surgical Research, Ft. Sam Houston, Texas; Brooke Army Medical Center, San Antonio, Texas; United States Army Institute of Surgical Research, San Antonio, Texas; AZ Burn Center at Valleywise Health, Cave Creek, Arizona; UT Southwestern, Dallas, Texas; University of Washington, Seattle, Washington; United States Institute of Surgical Research, San Antonio, Texas

## Abstract

**Introduction:**

Initial fluid infusion rates for resuscitation of burn injuries typically use formulas based on patient weight and total body surface area (TBSA) burned. However, the impact of this rate on overall resuscitation volumes and outcomes have not been extensively studied. The purpose of this study was to determine the impact of initial fluid rates on 24 hour volumes and outcomes using the Burn Navigator (BN).

**Methods:**

The BN database is composed of 300 patients with > 20% TBSA, >40kg that were resuscitated utilizing the BN. Four study arms were analyzed based on the initial formula – 2ml/kg/TBSA, 3 ml/kg/TBSA, 4 ml/kg/TBSA or the Rule of Ten. Total fluids infused at 24 hours after admission were compared as well as resuscitation-related outcomes.

**Results:**

A total of 296 patients were eligible for analysis. Higher starting rates (4 ml/kg/TBSA) resulted in significantly higher volumes at 24 hours (5.2 + 2.2 ml/kg/TBSA) than lower rates (2 ml/kg/TBSA resulted in 3.9 + 1.4 ml/kg/TBSA). No shock was observed in the high resuscitation cohort, whereas the lowest starting rate exhibited a 12% incidence, lower than both the Rule of Ten and 3 ml/kg/TBSA arms. There was no difference in 7-day mortality across groups.

**Conclusions:**

Higher initial fluid rates resulted in higher 24-hour fluid volumes. The choice of 2ml/kg/TBSA as initial rate did not result in increased mortality or more complications. An initial rate of 2ml/kg/TBSA is a safe strategy.

**Applicability of Research to Practice:**

Utilizing a lower starting fluid rate is a safe strategy and should considered after severe burn injury.

